# Analysis of the risk of complications during pregnancy in pregnant women with assisted reproductive technology: a retrospective study using registry linkage from 2013 to 2018 in Shanghai, China

**DOI:** 10.1186/s12884-022-04846-1

**Published:** 2022-06-28

**Authors:** Mulan He, Xiaoxi Sun, Chunfang Wang, Yilun Sui

**Affiliations:** 1 Obstetrics and Gynecology Hospital of Fudan University, 352#, Dalin Road, Shanghai, 200011 China; 2 Shanghai JiAi Genetics & IVF Institute, Shanghai, 200011 China; 3 Key Laboratory of Female Reproductive Endocrine Related Diseases, Shanghai, 200011 China; 4 Vital Statistical Department, Institute of Health Information, Shanghai Municipal Center for Disease Control and Prevention, Shanghai, China

**Keywords:** Assisted reproductive technology, Perinatal outcome, Pregnancy-induced hypertension, Frozen-thawed blastocyst transfer, Registry linkage study

## Abstract

**Background:**

To evaluate the differences in pregnancy outcomes between assisted reproductive technology (ART) patients and natural pregnant women in Shanghai, China in the past 6 years objectively. And to assess the feasibility of the research method of registry-database linkage in mainland China.

**Methods:**

This retrospective study was conducted using registry-database linkage. A total of 8102 pregnancies with ART and 8096 parturients with spontaneous conception (SC) from 10 reproductive centers and 111 hospitals composed our retrospective study. The primary outcomes were the rates of obstetric complications (pregnancy-induced hypertention [PIH], gestational diabetes mellitus [GDM], placenta previa, mode of delivery, preterm birth [PTB], low birth weight [LBW], and macrosomia). The prenatal outcomes were compared between ART and SC parturients, frozen-thawed embryo transfer (FET) and fresh embryo transfer, and in vitro fertilization (IVF) and intracytoplasmic sperm injection (ICSI). We calculated odds ratios (ORs) and 95% confidence intervals (CIs).

**Results:**

The final matching rate of the target population was 92% by using registry linkage. ART resulted in a higher rate of multiple birth, PTB, LBW, cesarean section, placenta previa and GDM compared with SC in the singleton cohort. In ART patients, pregnant women with FET had a significantly higher risk of PIH than those with fresh embryo transfer (14.1% Vs 9.3%, AOR1.528, 95% CI 1.303–1.793), but there was no difference between IVF and ICSI. FET is also related to the severity of PIH.

**Conclusions:**

ART increased the rate of complications during pregnancy, the risk and severity of PIH in patients with FET was higher than that in patients with fresh embryo transfer. The registry-database linkage study is an objective and feasible research method in mainland China.

**Supplementary Information:**

The online version contains supplementary material available at 10.1186/s12884-022-04846-1.

## Background

There are tens of thousands of infertile women all over the world get successful pregnancy by assisted reproductive technology (ART) every year. In the past, reproductive experts focused on how to improve the clinical pregnancy rate and cumulative live birth rate [1–3]. However, some non-physiological interventions in ART process, especially the use of super physiological dose hormone drugs, may affect the overall environment of pregnancy, interfere with gametogenesis or embryonic development, and have an adverse impact on the outcome of mother and newborn. With the increasing attention of experts to the health of ART mothers and newborns, many foreign studies have reported the perinatal outcomes of ART patients [4–6].

The prevalence of infertility in China had exceeded 15.5% in 2010 [7]. At present, the total number of cycles has exceeded 1.15 million cycles in vitro fertilization per year in Chinese mainland, and the number of offspring born with ART has exceeded 300,000 every year [8]. The success rate is basically close to the level of developed countries in Europe and the United States [9]. However, what does not match this situation is that China lacks a systematic, integrated, and standardized ART database [10]. Therefore, there are few articles in the database about the maternal and infant outcomes of Chinese mainland’s ART patients, and most of the results are from telephone follow-up, which obviously lacks objectivity.

In the present paper, we conducted a retrospective cohort study, attempted to match the treatment status and pregnancy outcome of ART patients by integrating the existing independent database in Shanghai, China. On the one hand, this study can objectively evaluate the differences in pregnancy outcomes between ART patients and natural pregnant women in Shanghai, China in the past few years. On the other hand, it can evaluate the feasibility of the research method of registry-database integration, and provide reference for relevant government departments to establish the ART registration and follow-up database in the future.

## Methods

### Data sources

This is a multicenter retrospective cohort study. The study population included women who underwent assisted reproduction treatment from January 2013 to December 2018 in 10 reproductive centers and delivered babies in 111 hospitals in Shanghai, China. The 10 fertility centers, scattered in six districts of Shanghai, include three reproductive medicine departments in specialized gynecology and obstetrics hospitals and seven in triple A comprehensive hospitals. Data collected at the fertility center included patient ID number (social security number), infertility factors, history of previous diseases and surgeries, number of ART cycles, and information on ART treatment including ovulation induction protocol, in vitro fertilization method, embryo development period in vitro, frozen or fresh embryo transfer, number of embryos transferred, and date of transfer. Finally, a total of 8102 patients with Shanghai household registration or long-term resident in Shanghai with complete data were collected.

The control group was comprised of Shanghai registered women with spontaneous conception (SC) and delivery in the Obstetrics and Gynecology Hospital of Fudan University from 2013 to 2018. According to the age of patients in the ART group (aged 23–45 years), 8096 parturients with complete case histories were 1:1 propensity score matched on age with a caliper width of 0.05 times the standard deviation of the propensity score.

### Data linkage

Two registry database links were used to match the primary outcome date that we wanted to study. A total of 12,017 ART patients’ information was collected from 10 fertility centers. The first database linkage was based on the ID number of patients and the date of transplantation. A total of 13,941 offspring of 11,228 ART patients were matched with birth information from Vital Statistical Department, Institute of Health Information, Shanghai Municipal Center for Disease Control and Prevention (CDC) and the data collected included: date of delivery, sex of newborn, gestational age at birth, birth weight, number of pregnancies, number of births, single or twin, delivery method, delivery hospital, malformation, type of malformation and ICD 10 (International Classification of Diseases 10th Edition) code.

**Fig. 1:**
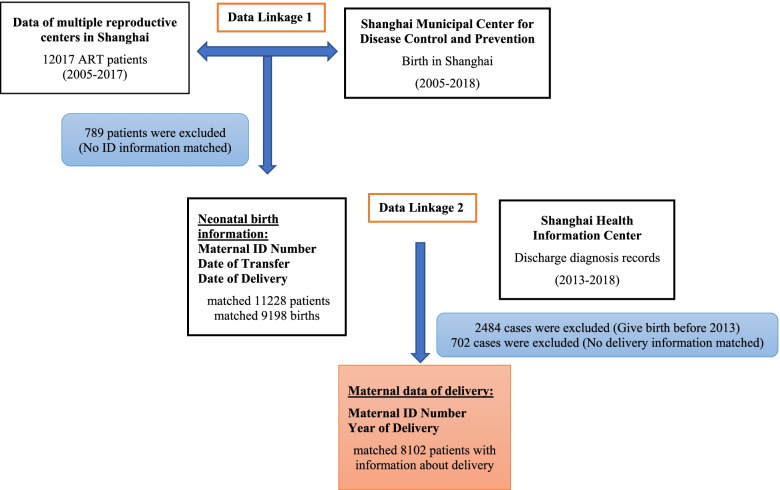
This flow chart shows the registration database involved in this study, the date linkage procedure, and the number of cases matched

The second registry database linkage was matched with the inpatient medical records of Shanghai Health Information Center through the mother’s ID number and delivery date, and a total of 8102 cases of the above-mentioned patients were matched with the information of the inpatient medical records. The matched information includes: discharge diagnosis (disease name and ICD 10 code), surgical procedure name and surgical procedure code. Since January 2013, the hospitalization history information of all registered hospitals in Shanghai has been uploaded to the database of the Health Information Center, so this link only matches the hospitalization information of patients in 2013 and later. (Fig. 1).

### Variables and primary outcome date

Since this was a retrospective study, the diagnosis of pregnancy outcome came from 111 hospitals, and the diagnostic criteria were not quite certain, we used the ICD10 code to match the disease. The primary obstetric complications were pregnancy-induced hypertension (PIH, ICD-10: O13.× 00, O13.× 01); preeclampsia (PE, ICD-10: O14.000, O14.900), severe preeclampsia (sPE, ICD-10: O14.100), eclampsia (ICD-10: O15.000, O15.001, O15.100, O15.101, O15.200, O15.201, O15.900), HELLP (hemolysis, elevated liver enzymes, and low platelets) syndrome (ICD-10: O14.200), except for chronic hypertension (ICD-10: O10, O11); gestational diabetes mellitus (GDM, ICD-10: O24.400), except for pre-existing diabetes (ICD-10: O24.0, O24.1, O24.2, O24.3, O24.9); placenta previa (ICD-10: O44.000, O44.001, O44.002, O44.003, O44.100, O44.101, O44.102, O44.103); rate of cesarean (including emergency cesarean and planned cesarean). Perinatal data including newborn’s birth weight, macrosomia (birth weight > 4000 g), low birth weight (LBW, birth weight < 2500 g), very low birth weight (VLBW, birth weight < 1500 g); preterm birth (PTB, birth earlier than gestation week 37), very / extremely preterm birth (birth earlier than gestation week 32). The additional parameters were included in the final analysis: maternal age; parity (primiparous: no previous births); gestational age at delivery (in weeks). The primary analysis focused on the comparison of hypertensive outcomes by types of maternal fertility treatment.

### Statistical analysis

Characteristics of the study population were described as number (percentage) for categorical variables and mean ± standard deviation (SD) for continuous variables. Student t test or Mann-Whitney U test was used to ascertain the significance of differences between mean values of 2 continuous variables. Chi-square test or Fisher exact test was performed to test for differences in proportions of categorical variables between groups. Regression modeling to predict factors contributing to PIH was performed with repeated measures multivariable logistic regression techniques, which accounted for possible confounders including patient factors (age, parity, number of cycles and infertility factors), treatment factors (FET, ICSI, period of transferred embryos and number of embryos transferred), and multiple pregnancies. Odds Ratios (OR) are presented with 95% Confidence Intervals (CI). All data was tested at the two-sided and Significance level was set at the < 0.05. Data analysis was done using IBM SPSS Statistics for Windows, Version 23.0 (IBM Crop., Armonk, NY, USA).

### Ethical approval

The study was approved by the ethics committees at the Shanghai Ji Ai Genetics and IVF Institute and other reproductive centers. A data safety monitoring board was established to oversee the study. All data entry, data management, and analyses were coordinated or performed at the Shanghai Scientific Association of Better Birth and Better Upbringing, which was the data-coordinating center for this study. In consideration of the anonymous nature of the final dataset, it is not possible to publicize results to individual study participants; instead, results will be shared with fertility practitioners and reproductive centers through the Shanghai Municipal Health Commission. There was no commercial support for this study.

## Results

12,017 ART patients were connected to the Shanghai CDC database through their ID number, and 789 patients who did not match their ID information were excluded. A total of 11,288 patients and 13,941 newborns were matched to the basic information of their birth (matching rate 93.9%). Then, the ID information and delivery date of the 11,288 patients were connected to the database of the Shanghai Health Information Center, and 2484 cases of delivery before 2013 were excluded, and 702 cases of delivery after 2013 were excluded from the database of 8804 cases of delivery without hospitalization information. In the end, 8102 patients’ delivery outcome information was obtained as the ART group for statistical analysis (matching rate 92.0%). A total of 39,462 patients with Shanghai household registration who delivered in Obstetrics and Gynecology Hospital of Fudan University in Shanghai from 2013 to 2018 were matched 1:1 with the ART group according to the age of the mothers. The delivery outcome information of a total of 8096 patients was used as the control group for statistical analysis. (Characteristics of ART pregnant women see Additional file 1).

Statistical analysis and comparison of pregnancy outcomes and neonatal conditions in the ART group and the control group showed that the incidence of twins in the ART group was significantly higher than that in the natural pregnant women (26.4% Vs 1.1%, *p* < 0.001). The incidence of preterm birth (< 37 weeks and < 32 weeks) was higher in ART patients than in controls (19.5% Vs 6.2 and 1.8% Vs 0.5%, *p* < 0.001). Excluding multiple pregnancies, the incidence of preterm delivery was significantly lower in the singleton cohort, but still higher than in the control cohort (7.0% Vs 5.6% *p* = 0.001, and 0.9% Vs 0.4% *p* = 0.002). The neonates in ART group had lower birth weight than those in control group (3086.3 ± 650.0 g Vs 3306.0 ± 478.0 g, *p* < 0.001), and the proportion of low birth weight and very low birth weight infants were significantly higher than those in control group (18.1% Vs 4.6 and 1.9% Vs 0.4%, *p* < 0.001). The proportion of macrosomia was lower than that of the control group (5.5% Vs 6.2%, *p* = 0.071). The proportion of LBW and VLBW in the single-child cohort was significantly lower, but still significantly higher than in the control cohort (4.4% Vs 3.9% *p* < 0.001, and 0.7% Vs 0.3% *p* = 0.007). In terms of maternal pregnancy outcome, the rate of cesarean section was significantly higher in the ART group than in the control group (76.3% Vs 50.6% for all cohorts, 69.1% Vs 50.0% for single cohorts, *p* < 0.001). Patients in the ART group developed placenta previa during pregnancy (6.9% Vs 2.0% in all cohorts, 6.2% Vs 2.0% in singleton cohorts, *p* < 0.001) and gestational diabetes mellitus were significantly higher than those in the control group (22.3% Vs 17.4% in all cohorts, 21.9% Vs 17.4% in singleton cohorts, *p* < 0.001). In all cohorts, the incidence of PIH was higher in the ART group than in the control group (12.6% Vs 9.4%, *p* < 0.001), but there was no difference in the incidence of PIH after multiple pregnancies were excluded (9.7% Vs 9.3%, *p* = 0.420). (Table 1.)

**Table 1:** Comparison of pregnancy outcomes between ART pregnant women and spontaneously conceived women

Parameter	Full Cohort		***P*** value ^c^	Singletons Only		***P*** value
	ART^a^ (***n =*** 8102)	SC^b^ (***n =*** 8096)		ART (***n =*** 5960)	SC (***n =*** 8005)	
**Maternal age (years, mean (SD))**	32.4 ± 3.6	32.4 ± 3.6	0.997	32.7 ± 3.7	32.4 ± 3.7	**< 0.001**
**Primiparous** (**No.(%)**)	7420(91.6)	6013(74.3)	**< 0.001**	5734(96.2)	6012(75.1)	**< 0.001**
**Single/multiple births (No.(%))**			**< 0.001**	/	/	**/**
single	5960(73.6)	8005(98.9)				
twins	2135(26.4)	89(1.1)				
triplets	7(0.1)	2(0.0)				
**Gestational age (wk, mean (SD))**	37.7 ± 2.1	38.6 ± 1.5	**< 0.001**	38.4 ± 1.6	38.6 ± 1.4	**< 0.001**
< 32 weeks (No.(%))	143(1.8)	43(0.5)	**< 0.001**	52(0.9)	36(0.4)	**0.002**
< 37 weeks (No.(%))	1582(19.5)	505(6.2)	**< 0.001**	415(7.0)	450(5.6)	**0.001**
**Birth weight (g, mean (SD))**	3086.3 ± 650.0	3306.0 ± 478.0	**< 0.001**	3346.8 ± 497.7	3317.7 ± 465.4	**< 0.001**
< 1500 g (No.(%))	150(1.9)	30(0.4)	**< 0.001**	39(0.7)	25(0.3)	**0.007**
< 2500 g (No.(%))	1468(18.1)	374(4.6)	**< 0.001**	264(4.4)	312(3.9)	**< 0.001**
≥4000 g (No.(%))	445(5.5)	501(6.2)	**0.071**	445(7.5)	499(6.2)	**0.010**
**Obstetrical events (No.(%))**						
Cesarean section	6183(76.3)	4093(50.6)	**< 0.001**	4118(69.1)	4005(50.0)	**< 0.001**
Placenta previa	558(6.9)	161(2.0)	**< 0.001**	368(6.2)	159(2.0)	**< 0.001**
Hypertensive disorders	1017(12.6)	763(9.4)	**< 0.001**	578(9.7)	744(9.3)	0.420
Gestational Diabetes	1806(22.3)	1412(17.4)	**< 0.001**	1303(21.9)	1389(17.4)	**< 0.001**

^a^. *ART* Assisted reproductive technology

^b^. *SC* Spontaneously conceived pregnancy

^c^. Date are statistically significant at *p* < 0.05

We grouped ART patients according to different treatment regimens and compared the differences in pregnancy outcomes between the groups. 8102 ART patients were transplanted with different embryo types, including 2589 patients with Fresh embryos (Fresh group) and 5513 patients with freeze-thaw embryos (FET group). Comparing two groups of pregnant women, the results indicated that pregnant women with freeze-thaw embryo transfer had a significantly higher incidence of PIH than those with fresh embryo transfer (14.1% Vs 9.3%, *p* < 0.001). Analysis of the single-embryo cohort alone (4071 freeze-thawing embryos and 1889 fresh embryos) showed that the rate of hypertensive disease during pregnancy was still significantly higher in patients receiving FET than in patients receiving fresh embryos (11.0% Vs 6.9%, *p* < 0.001) (Table 2). In addition, fertilized eggs were obtained from ART patients using different methods of in vitro fertilization, including 5144 patients with IVF and 2958 patients with ICSI. Comparison of pregnancy outcomes between IVF group and ICSI group showed that there were no significant differences in the incidence of placenta previa, PIH and GDM (*p* > 0.05) (Table 3).

**Table 2:** Comparison of pregnancy outcomes between fresh and frozen embryo transfer cycles

Parameter	Full Cohort		***P*** value^a^	Singletons Only		***P*** value
	Frozen Transfers (***n =*** 5513)	Fresh Transfers (***n =*** 2589)		Frozen Transfers (***n =*** 4071)	Fresh Transfers (***n =*** 1889)	
**Maternal age (years, mean (SD))**	32.5 ± 3.7	32.3 ± 3.6	0.046	32.7 ± 3.8	32.5 ± 3.7	0.023
**Gestational age (wk, mean (SD))**	37.7 ± 2.2	37.8 ± 2.0	0.020	38.4 ± 1.7	38.5 ± 1.5	0.008
< 32 weeks (No.(%))	106(1.9)	32(1.2)	0.026	41(1.0)	10(0.5)	0.062
< 37 weeks (No.(%))	1124(20.4)	458(17.7)	0.004	314(7.7)	105(5.6)	0.002
**Birth weight (g, mean (SD))**	3142.0 ± 614.7	3106.3 ± 591.7	0.013	3353.5 ± 511.4	3329.6 ± 467.1	0.076
< 1500 g (No.(%))	95(1.7)	32(1.2)	0.098	32(0.8)	7(0.4)	0.064
< 2500 g (No.(%))	751(13.6)	370(14.3)	0.432	199(4.9)	64(3.4)	0.009
≥4000 g (No.(%))	316(5.7)	129(5.0)	0.163	316(7.8)	129(6.8)	0.202
**Obstetrical events (No.(%))**						
Cesarean section	4336(78.7)	1847(71.3)	**< 0.001**	2940(72.2)	1178(62.4)	**< 0.001**
Placenta previa	374(6.8)	184(7.1)	0.592	251(6.2)	117(6.2)	0.966
Hypertensive disorders	775(14.1)	242(9.3)	**< 0.001**	448(11.0)	130(6.9)	**< 0.001**
Gestational Diabetes	1248(22.6)	558(21.6)	0.274	897(22.0)	406(21.5)	0.638

^a^. Date are statistically significant at *p* < 0.05

**Table 3:** Comparison of pregnancy outcomes between IVF and ICSI pregnancies

Parameter	Full Cohort		***P*** value^c^	Singletons Only		***P*** value
	IVF^a^ (***n =*** 5144)	ICSI^b^ (***n =*** 2958)		IVF (***n =*** 3697)	ICSI (***n =*** 2263)	
**Maternal age (years, mean (SD))**	32.5 ± 3.6	32.4 ± 3.7	0.169	32.7 ± 3.7	32.6 ± 3.8	0.427
**Gestational age (wk, mean (SD))**	37.7 ± 2.2	37.8 ± 2.0	0.003	38.5 ± 1.7	38.4 ± 1.6	0.925
< 32 weeks (No.(%))	94(1.8)	44(1.5)	0.255	33(0.9)	18(0.8)	0.693
< 37 weeks (No.(%))	1050(20.4)	532(18.0)	0.008	266(7.2)	153(6.8)	0.525
**Birth weight (g, mean (SD))**	3116.3 ± 614.1	3155.8 ± 595.8	0.005	3346.2 ± 497.4	3345.8 ± 499.0	0.978
< 1500 g (No.(%))	87(1.7)	40(1.4)	0.235	24(0.6)	15(0.7)	0.949
< 2500 g (No.(%))	749(14.6)	372(12.6)	0.012	166(4.5)	97(4.3)	0.710
≥4000 g (No.(%))	275(5.3)	170(5.7)	0.450	275(7.4)	170(7.5)	0.916
**Obstetrical events (No.(%))**						
Cesarean section	3936(76.5)	2247(76.0)	0.609	2543(68.8)	1575(69.6)	0.655
Placenta previa	361(7.0)	197(6.7)	0.540	231(6.2)	137(6.1)	0.762
Hypertensive disorders	636(12.4)	381(12.9)	0.499	340(9.2)	238(10.5)	0.095
Gestational Diabetes	1136(22.1)	670(22.7)	0.555	805(21.8)	498(22.0)	0.834

^a^. IVF, in vitro fertilization

^b^. ICSI, intracytoplasmic sperm injection

^c^. Date are statistically significant at *p* < 0.05

We further analyzed the risk factors of PIH in ART patients during pregnancy. In terms of patient factors, age was a high-risk factor for hypertensive disease during pregnancy (1.038 [95%CI 1.018–1.058]). Only male-induced infertility was a protective factor, but we separately analyzed female infertility factors, including endometriosis, ovulation disorders and tubal infertility, and none of them were suggested as risk factors for hypertension during pregnancy. For ART treatment, FET alone significantly increased the risk of hypertensive disease during pregnancy (AOR 1.528 [95%CI 1.303–1.793]). Other interventions such as ICSI, embryonic development stage, and number of embryos transferred were not high-risk factors. In addition, logistic regression results showed that multiple pregnancies significantly increased the risk of PIH (AOR 2.536 [95%CI 2.204–2.917]). (Table 4) The above results showed that only FET influenced the occurrence of hypertension during pregnancy among ART treatment factors. Therefore, we analyzed whether FET had any difference or influence on the occurrence of hypertension during pregnancy with different severity. The results suggest that, pregnant women with FET were at significantly increased risk for PIH (AOR 1.370 [95% CI 1.092–1.718]), mild preeclampsia (AOR 1.427 [95% CI 1.102–1.850]), and severe preeclampsia, including HELLP syndrome (AOR 2.170 [95% CI 1.583–2.975]). (Table 5).

**Table 4:** The risk of hypertensive disorders in ART pregnancies ^a^

Variable	Risk of PIH^b^ (Crude OR) ^c^			Risk of PIH (Adjusted OR) ^d^		
	OR	95% CI	***P*** value	OR	95% CI	***P*** value
**Patient factors**						
Patient age (years)	1.023	1.005–1.042	**0.011**	1.038	1.018–1.058	**< 0.001**
Primiparous	1.060	0.789–1.423	0.701	1.221	0.903–1.651	0.194
No. of cycles						
1–2	ref	/	/	ref	/	/
≥3	1.215	0.963–1.534	0.100	1.116	0.877–1.422	0.372
Male factor only	0.675	0.545–0.837	**< 0.001**	0.714	0.558–0.913	**0.007**
Endometriosis diagnosis	0.640	0.344–1.191	0.159	0.681	0.361–1.287	0.237
Ovulatory disorder (mainly PCOS)	1.330	0.989–1.788	0.059	1.206	0.879–1.653	0.239
Tubal diagnosis	0.969	0.850–1.105	0.640	0.968	0.832–1.127	0.677
**Treatment factors**						
Frozen embryo transfer	1.586	1.362–1.848	**< 0.001**	1.528	1.303–1.793	**< 0.001**
ICSI	1.048	0.915–1.200	0.499	1.141	0.986–1.321	0.077
Period of transferred embryos						
Day 2–3	ref	/	/	ref	/	/
Day 4	0.941	0.400–2.212	0.889	0.940	0.731–1.209	0.631
Day 5–6	1.190	0.936–1.513	0.154	1.111	0.452–2.727	0.819
No. of embryos transferred						
1	0.813	0.591–1.120	0.206	1.407	0.875–2.263	0.159
2	ref	/	/	ref	/	/
3	0.771	0.483–1.230	0.274	1.532	0.861–2.724	0.147
Multiple pregnancies	2.400	2.096–2.749	**< 0.001**	2.536	2.204–2.917	**< 0.001**

^a^. The research objects in this table are all ART patients, *n =* 8102

^b^. PIH, pregnancy-induced hypertention

^c^. All odds ratio are for the risk of PIH from logistic regression

^d^. Adjusted for patient factors (age, parity, number of cycles and infertility factors), treatment factors (frozen embryo transfer, ICSI, period of transferred embryos and number of embryos transferred), andmultiple pregnancies

**Table 5:** The risk of different severity of hypertensive disease during pregnancy in the FET compared with the fresh embryo transfer

Severity	FET versus Fresh ^a^								
	n	B	***P*** value	Crude OR^b^	95% CI	B	***P*** value	Adjusted OR ^c^	95% CI
**No hypertensive disorders**	7085	ref	/	/	/	ref	/	/	/
**PIH**	413	0.343	**0.003**	1.409	1.126–1.762	0.315	**0.006**	1.370	1.092–1.718
**Mild pre-eclampsia**	332	0.372	**0.004**	1.450	1.125–1.870	0.356	**0.007**	1.427	1.102–1.850
**Severe pre-eclampsia & HELLP**^d^	272	0.788	**< 0.001**	2.199	1.612–3.001	0.775	**< 0.001**	2.170	1.583–2.975

^a^. The research objects in this table are all FET (*n =* 5513) and fresh transfers (*n =* 2589)

*FET* Frozen-thawed embryo transfer

^b^. All odds ratio are for the severity of PIH from multinomial logistics regression

^c^. Adjusted for maternal age, only male infertility, frozen embryo transfer, ICSI, multiple pregnancies

^d^. HELLP, hemolysis, elevated liver enzymes, and low platelets

## Discussion

A retrospective cohort study was conducted on the perinatal outcomes of pregnant women who had undergone ART from 2013 to 2018 in Shanghai, China by means of registry-database linkage, and the matching rate of the target population was 92%. The results showed that ART resulted in a higher rate of multiple birth, PTB, LBW, cesarean section, placenta previa and GDM compared with spontaneous conception, not only in the full cohort, but also in the singletons cohort. As for PIH, the incidence of ART was higher than spontaneous conception in the full cohort, but there was no difference in the singleton cohort. In the singleton cohort, the rates of PTB, LBW, cesarean section, and PIH in frozen embryo transfer women were higher than those in fresh embryo transfer, and there was no difference between IVF and ICSI. Multivariate regression analysis showed that after excluding the influence of other factors, the risk of PIH in patients with FET was still increased. Moreover, FET is also related to the severity of pregnancy induced hypertension in our cohort.

In terms of perinatal outcomes of ART patients, many foreign reproductive researches have been reported in recent years many, most of which are retrospective [4–6]. The consistent results are that the incidence of multiple births and preterm birth after ART is significantly increased, and the corresponding complications are also increased [11, 12]. Our results showed that the rate of multiple birth was 24 times higher in the ART group than in the SC group. In addition, of all the 12,017 patients we collected, only 592 (4.9%) had 1 embryo transferred, 10,525 (87.6%) had 2 embryos transferred, and even 900 (7.5%) had 3 embryos transferred. With foreign experts advocating elective single embryo transfer (eSET) to reduce the incidence of multiple pregnancy and protect the safety of mother and child. In November 2018, Shanghai municipality issued a notice on The Regulations for Human Assisted Reproductive Technology in Shanghai, which clearly suggested the total number of embryos transferred per cycle shall not exceed 2, and no more than one embryo should be transferred during the first cycle. Even if only comparing the pregnancy outcomes of singleton offspring, the results of this study suggest that the incidence of preterm birth and very preterm birth in ART singleton infants is significantly higher than that in natural pregnancy offspring, and the incidence of LBW and VLBW is also significantly higher than that in natural pregnancy offspring. These results are consistent with the results reported in several previous literatures [13, 14].

The most common complications of pregnancy are pregnancy induced hypertension syndrome (PIH) and gestational diabetes mellitus (GDM). A meta-analysis showed that ART are linked to increased risk of GDM [15]. As GDM pregnant women, the patients after ART treatment have a higher risk of adverse pregnancy outcomes [16, 17]. The results of this study are consistent with those reported. As for PIH, prior literatures have reported that ART therapy is correlated with the occurrence of PIH to a certain extent [18, 19]. In the current study, the incidence of PIH in ART patients increased only in the full cohort, and was not significantly different in the singleton cohort compared with natural pregnancy. This may indicate that the high incidence of PIH in ART patients is mainly related to the high incidence of multiple pregnancies [19]. Another possible reason is that the control group of pregnant women in this study came from a triple A obstetrics and gynecology hospital. It can be seen from the results that the incidence of PIH in the singleton cohort of SC group is as high as 9.3%, which is higher than the incidence reported in the literature [20].

Since it is still controversial whether the influence of maternal infertility factors or the process of art treatment plays a leading role in the increase of the risk of adverse pregnancy outcomes [21, 22], we compared the pregnancy outcomes of ART patients receiving different treatment protocols, and the results suggested that different in vitro fertilization methods had no effect on pregnancy outcomes. However, frozen embryo transfer was a risk factor for PIH, with an adjusted OR of 1.528 (95%CI: 1.303–1.793). After controlling for maternal age, male infertility factors only, and multiple pregnancies, the risk of PIH in patients using FET treatment was still higher than that in patients using fresh cycles. In addition, the more severe the PIH, the higher the adjusted OR value of FET patients. In fact, with the increasing use of FET in clinical practice, relevant studies have found that the risk of PIH/preeclampsia after FET treatment is higher than that after fresh embryo transfer [23, 24]. A multicenter randomized controlled trial in China found that the risk of preeclampsia after FET was increased in patients with PCOS [25], but there was no difference in the infertile patients with normal ovulation [26].

However, the specific mechanism is still unclear, which may be related to the fact that FET is more prone to abnormal placental anatomy and vascular pathological changes [27]. In addition, a prospective cohort study found that pregnant women using the programmed FET cycle had a higher risk of PIH than those using natural cycle [28]. Because ovulation is inhibited in patients with programmed FET cycle, these pregnant women lack endogenous corpus luteum (CL), and need progesterone support after transplantation. It was found that the number of corpus luteum in early pregnancy of IVF pregnant women may be related to the risk of PIH / preeclampsia [29–31]. This hypothesis is biologically plausible because CL produces hormones such as estrogen, progesterone, relaxin, and vascular endothelial growth factor (VEGF) [32–34]. As relaxin and VEGF cannot be supplemented by drugs, the lack of these vasoactive substances in the programmed FET cycle may lead to the initial placental formation disorders or the decreased maternal circulation adaptation [30, 35, 36]. However, clinical observational studies cannot exclude the influence of other confounding factors, so its specific mechanism needs further research. In this study, FET patients could not distinguish between the programmed cycle and the natural cycle, because relevant information was not collected during the first part of data collection, so no further subgroup analysis was performed.

In addition, the difference of the pregnancy outcomes between different fertilization methods (IVF and ICSI) in this study was not significant. In reviewing the literature, the effect of ICSI on maternal pregnancy outcome remains controversial. The current consensus is that ICSI offspring have a higher risk of birth defects than normal pregnancy offspring and IVF offspring [37]. The results of this study also revealed that ART was associated with significantly higher odds of PTB, LBW, cesarean section, and placenta previa. These results are in line with those of previous studies [38–40].

The main advantage of this study is that it uses the high-quality registry data of the government health department to search and match the pregnancy outcomes and newborns of the subjects. The reliability and accuracy are significantly higher than the pregnancy outcomes obtained by telephone follow-up of reproductive centers. According to a report on 70 years of women’s reproductive, maternal, newborn, child, and adolescent health in China, by the end of 2019, there were 517 medical institutions approved to carry out human assisted reproductive technology in China [10]. However, in the follow-up of ART patients, patients are reluctant to cooperate due to the privacy of patients involved, and there is still no national ART reporting system with integrated information, leading to a high rate of lost follow-up of ART patients after childbirth in mainland China. Several high-quality multicenter prospective cohort studies are still in their infancy, the number of cases is still small [26]. Therefore, there are few studies and reports on maternal pregnancy outcomes after ART treatment in China in the past 20 years. The pregnancy outcomes involved in some studies are obtained from patients by the staff of the reproductive center through telephone follow-up. They are not professional in disease name and severity classification, and the data on birth weight and gestational week are not accurate enough, resulting in the results obtained by this survey method having a certain subjective impact and inaccurate. The research method of this paper can avoid this problem, make the results more accurate and have more reference value. At the same time, because it is a group survey, the result report does not involve the patient’s personal privacy.

This study has some limitations. First, the data on ART treatment are from 10 different reproductive centers in Shanghai. We can’t assess whether the specific operation processes are completely consistent. We can only consider that they are all qualified reproductive centers, which should be operated according to the specified operation specifications, and the sample size is sufficient, which can reduce this part of bias. Secondly, the patients in this study delivered in 111 hospitals in different districts and counties of Shanghai, and the standard of disease diagnosis and the control of medical history quality cannot be monitored. Therefore, we did not use the name of disease diagnosis for screening, but chose to use the ICD-10 code of disease to unify the standard, but we still cannot completely avoid the inaccurate clinical diagnosis. Third, our study is retrospective. When collecting the basic information of patients, we lack some possible interfering factors, such as the patient’s race, occupation, pre-pregnancy BMI, smoking, alcohol abuse, etc. Most medical histories do not contain relevant information and are therefore excluded from the statistics. All the cases included in our study were female with household registration in Shanghai, so the influence of different races, different family backgrounds and dietary habits was relatively reduced, but the influence of pre-pregnancy BMI on the results could not be avoided. Finally, the cases in the natural pregnancy group came from the affiliated obstetrics and Gynecology Hospital of Fudan University, which is a AAA specialized hospital in Shanghai. District hospitals will choose to refer to the hospital when they encounter unmanageable twins, pregnancy complications and other high-risk pregnant women. Therefore, the proportion of natural pregnancy twins and pregnancy complications in this hospital will be higher than the average level in Shanghai. However, the incidence of ART group was still significantly higher than that of the control group. Considering that compared with the average level of the city, it is expected that the OR value will be higher.

Our study and literature strongly suggest that FET increase the risk of PIH/preeclampsia, but the specific influencing factors are not clear so far. Although some studies have reported that the use of hormone in programmed FET cycles leads to endogenous luteal deficiency, the specific mechanism remains to be further studied. As the influence of China’s three-child policy, the utilization of FET is more and more widely, increasing number of elderly women with low fertility will be affected. This is of great significance for conducting relevant basic research to explore its mechanism, and standardizing the ART treatment process to avoid possible pathogenic risks and improve the safety of ART.

## Conclusions

In conclusion, the matching rate of the target population can be as high as 92% by using registry linkage, indicating that this is an objective and feasible research method. In addition, the retrospective results on outcome of pregnancy after ART showed that ART increased the rate of complications during pregnancy, the risk and severity of PIH in patients with FET was higher than that in patients with fresh embryo transfer. Although this is a retrospective study, there are some limitations, and the results of our study are like existing literature reports. However, this study is the first attempt to use the method of registration database link in Chinese mainland. Therefore, the results of this study are objective and accurate, which can well reflect the safety of ART used in Shanghai in the past few years, and fill in the lack of literature in the database. In addition, our results have important reference value for promoting the establishment of national ART reporting system to collect case data and results in Mainland China.

## Supplementary Information

**Additional file 1 Supplemental Table 1.** Characteristics of ART pregnant women

## Data Availability

The datasets used and/or analyzed during the current study are available from the corresponding author on reasonable request.

## Abbreviations

ART: Assisted reproductive technology
CDC: Center for Disease Control and Prevention
CI: Confidence intervals
CL: Corpus luteum
FET: Frozen-thawed embryo transfer
GDM: Gestational diabetes mellitus
ICD: International Classification of Diseases
IVF: In vitro fertilization
ICSI: Intracytoplasmic sperm injection
LBW: Low birth weight
OR: Odds ratios
PCOS: Polycystic ovary syndrome
PIH: Pregnancy-induced hypertension
PE: Preeclampsia
PTW: Preterm birth
SD: Standard deviation
sPE: Severe preeclampsia
VLBW: Very low birth weight
VEGF: Vascular endothelial growth factor

## References

[1] Drakopoulos P, Blockeel C, Stoop D, Camus M, de Vos M, Tournaye H (2016). Conventional ovarian stimulation and single embryo transfer for IVF/ICSI. How many oocytes do we need to maximize cumulative live birth rates after utilization of all fresh and frozen embryos. Hum Reprod.

[2] Legro RS, Brzyski RG, Diamond MP, Coutifaris C, Schlaff WD, Casson P (2014). Letrozole versus clomiphene for infertility in the polycystic ovary syndrome. N Engl J Med.

[3] Polyzos NP, Drakopoulos P, Parra J, Pellicer A, Santos-Ribeiro S, Tournaye H (2018). Cumulative live birth rates according to the number of oocytes retrieved after the first ovarian stimulation for in vitro fertilization/intracytoplasmic sperm injection: a multicenter multinational analysis including ∼15,000 women. Fertil Steril.

[4] Berntsen S, Söderström-Anttila V, Wennerholm UB, Laivuori H, Loft A, Oldereid NB (2019). The health of children conceived by ART: 'the chicken or the egg?'. Hum Reprod Update.

[5] Raatikainen K, Kuivasaari-Pirinen P, Hippeläinen M, Heinonen S (2012). Comparison of the pregnancy outcomes of subfertile women after infertility treatment and in naturally conceived pregnancies. Hum Reprod.

[6] Zhang WY, von Versen-Höynck F, Kapphahn KI, Fleischmann RR, Zhao Q, Baker VL (2019). Maternal and neonatal outcomes associated with trophectoderm biopsy. Fertil Steril.

[7] Zhou Z, Zheng D, Wu H, Li R, Xu S, Kang Y (2018). Epidemiology of infertility in China: a population-based study. BJOG..

[8] Lane R (2021). Jun Zhu: China's maternal and child health surveillance supremo. Lancet..

[9] Bai F, Wang DY, Fan YJ, Qiu J, Wang L, Dai Y (2020). Assisted reproductive technology service availability, efficacy and safety in mainland China: 2016. Hum Reprod.

[10] Qiao J, Wang Y, Li X, Jiang F, Zhang Y, Ma J (2021). A lancet commission on 70 years of women's reproductive, maternal, newborn, child, and adolescent health in China. Lancet..

[11] Premru-Srsen T, Bokal Vrtačnik E, Bizjak T, Verdenik I, Korošec S, Ban FH (2021). Preterm delivery risk in infertile women who conceived after reproductive surgery: natural conception versus IVF/ICSI. Hum Reprod.

[12] Scholten I, Chambers GM, van Loendersloot L, van der Veen F, Repping S, Gianotten J (2015). Impact of assisted reproductive technology on the incidence of multiple-gestation infants: a population perspective. Fertil Steril.

[13] Johnson KM, Hacker MR, Thornton K, Young BC, Modest AM (2020). Association between in vitro fertilization and ischemic placental disease by gestational age. Fertil Steril.

[14] Schieve LA, Meikle SF, Ferre C, Peterson HB, Jeng G, Wilcox LS (2002). Low and very low birth weight in infants conceived with use of assisted reproductive technology. N Engl J Med.

[15] Bosdou JK, Anagnostis P, Goulis DG, Lainas GT, Tarlatzis BC, Grimbizis GF (2020). Risk of gestational diabetes mellitus in women achieving singleton pregnancy spontaneously or after ART: a systematic review and meta-analysis. Hum Reprod Update.

[16] Kouhkan A, Baradaran HR, Hosseini R, Arabipoor A, Moini A, Pirjani R (2019). Assisted conception as a potential prognostic factor predicting insulin therapy in pregnancies complicated by gestational diabetes mellitus. Reprod Biol Endocrinol.

[17] Kouhkan A, Khamseh ME, Pirjani R, Moini A, Arabipoor A, Maroufizadeh S (2018). Obstetric and perinatal outcomes of singleton pregnancies conceived via assisted reproductive technology complicated by gestational diabetes mellitus: a prospective cohort study. BMC Pregnancy Childbirth.

[18] Lynch A, McDuffie R, Murphy J, Faber K, Orleans M (2002). Preeclampsia in multiple gestation: the role of assisted reproductive technologies. Obstet Gynecol.

[19] Wang YA, Chughtai AA, Farquhar CM, Pollock W, Lui K, Sullivan EA (2016). Increased incidence of gestational hypertension and preeclampsia after assisted reproductive technology treatment. Fertil Steril.

[20] Hutcheon JA, Lisonkova S, Joseph KS (2011). Epidemiology of pre-eclampsia and the other hypertensive disorders of pregnancy. Best Pract Res Clin Obstet Gynaecol.

[21] Luke B, Gopal D, Cabral H, Diop H, Stern JE (2016). Perinatal outcomes of singleton siblings: the effects of changing maternal fertility status. J Assist Reprod Genet.

[22] Pirtea P, de Ziegler D, Poulain M, Ayoubi JM (2020). Which key performance indicators are optimal to assess clinical management of assisted reproduction cycles. Fertil Steril.

[23] Luke B, Brown MB, Eisenberg ML, Callan C, Botting BJ, Pacey A (2020). In vitro fertilization and risk for hypertensive disorders of pregnancy: associations with treatment parameters. Am J Obstet Gynecol.

[24] Maheshwari A, Pandey S, Amalraj Raja E, Shetty A, Hamilton M, Bhattacharya S (2018). Is frozen embryo transfer better for mothers and babies? Can cumulative meta-analysis provide a definitive answer. Hum Reprod Update.

[25] Shi Y, Sun Y, Hao C, Zhang H, Wei D, Zhang Y (2018). Transfer of fresh versus frozen embryos in ovulatory women. N Engl J Med.

[26] Chen ZJ, Shi Y, Sun Y, Zhang B, Liang X, Cao Y (2016). Fresh versus frozen embryos for infertility in the polycystic ovary syndrome. N Engl J Med.

[27] Sacha CR, Harris AL, James K, Basnet K, Freret TS, Yeh J (2020). Placental pathology in live births conceived with in vitro fertilization after fresh and frozen embryo transfer. Am J Obstet Gynecol.

[28] Saito K, Kuwahara A, Ishikawa T, Morisaki N, Miyado M, Miyado K (2019). Endometrial preparation methods for frozen-thawed embryo transfer are associated with altered risks of hypertensive disorders of pregnancy, placenta accreta, and gestational diabetes mellitus. Hum Reprod.

[29] Conrad KP, Petersen JW, Chi YY, Zhai X, Li M, Chiu KH (2019). Maternal cardiovascular dysregulation during early pregnancy after in vitro fertilization cycles in the absence of a Corpus luteum. Hypertension..

[30] von Versen-Höynck F, Häckl S, Selamet Tierney ES, Conrad KP, Baker VL, Winn VD (2020). Maternal vascular health in pregnancy and postpartum after assisted reproduction. Hypertension..

[31] von Versen-Höynck F, Schaub AM, Chi YY, Chiu KH, Liu J, Lingis M (2019). Increased preeclampsia risk and reduced aortic compliance with in vitro fertilization cycles in the absence of a Corpus luteum. Hypertension..

[32] Conrad KP, Baker VL (2013). Corpus luteal contribution to maternal pregnancy physiology and outcomes in assisted reproductive technologies. Am J Phys Regul Integr Comp Phys.

[33] Johnson MR, Abdalla H, Allman AC, Wren ME, Kirkland A, Lightman SL (1991). Relaxin levels in ovum donation pregnancies. Fertil Steril.

[34] von Versen-Höynck F, Strauch NK, Liu J, Chi YY, Keller-Woods M, Conrad KP (2019). Effect of mode of conception on maternal serum Relaxin, creatinine, and sodium concentrations in an infertile population. Reprod Sci.

[35] Debrah DO, Novak J, Matthews JE, Ramirez RJ, Shroff SG, Conrad KP (2006). Relaxin is essential for systemic vasodilation and increased global arterial compliance during early pregnancy in conscious rats. Endocrinology..

[36] von Versen-Höynck F, Narasimhan P, Selamet Tierney ES, Martinez N, Conrad KP, Baker VL (2019). Absent or excessive Corpus luteum number is associated with altered maternal vascular health in early pregnancy. Hypertension..

[37] Wen J, Jie J, Ding C, Dai J, Yao L, Xia Y (2012). Birth defects in children conceived by invitro fertilization and intracytoplasmic sperm injection: a meta-analysis. Fertil Steril.

[38] Dayan N, Lanes A, Walker MC, Spitzer KA, Laskin CA (2016). Effect of chronic hypertension on assisted pregnancy outcomes: a population-based study in Ontario. Canada Fertil Steril.

[39] Luke B, Gopal D, Cabral H, Stern JE, Diop H (2017). Pregnancy, birth, and infant outcomes by maternal fertility status: the Massachusetts outcomes study of assisted reproductive technology. Am J Obstet Gynecol.

[40] Yang X, Li Y, Li C, Zhang W (2014). Current overview of pregnancy complications and live-birth outcome of assisted reproductive technology in mainland China. Fertil Steril.

