# Supplementary material for: Analysis of the risk of complications during pregnancy in pregnant women with assisted reproductive technology: a retrospective study using registry linkage from 2013 to 2018 in Shanghai, China
Source: BMC Pregnancy Childbirth. 2022 Jun 28;22:526. doi: 10.1186/s12884-022-04846-1 (PMC9241204; doi:10.1186/s12884-022-04846-1)
Supplement: Supplementary file 1 — Additional file 1 Supplemental Table 1. Characteristics of ART pregnant women [file 12884_2022_4846_MOESM1_ESM.docx]

**Supplemental table 1. Characteristics of ART pregnant women**

| **Characteristic & Profiles** | **2005-2012**^†^  **(N = 3963)** | **2013-2018**^†^  **（N=8054）** |
| --- | --- | --- |
| **Mother`s age at pregnancy (years; mean (SD))** | 32.4±3.8 | 32.5±3.7 |
| **Father`s age at pregnancy (years; mean (SD))** | 34.9±6.0 | 34.6±5.5 |
| **Primary infertility (No.(%))** | 2423 (61.1) | 5077 (63.0) |
| **Cause of infertility (No.(%))**  Male factor only  Any female factor  Tubal factor  Ovulatory disorder (mainly PCOS)  Endometriosis  Uterine factor  Unexplained  Mixed factors  Combined chromosomal abnormalities | 794 (20.0)  2389 (60.3)  100 (2.5)  82 (2.1)  10 (0.3)  77 (1.9)  510 (12.9)  52 (1.3) | 1084 (13.5)  3912 (48.6)  374 (4.7)  118 (1.5)  35 (0.4)  604 (7.5)  1922 (23.9)  94 (1.2) |
| **Source of sperm (No.(%))**  Ipsism  Epididymal sperm extraction  Testicular sperm extraction  Donor sperm | 3622 (91.4)  95 (2.4)  89 (2.2)  157 (4.0) | 7647 (94.9)  85 (1.1)  83 (1.0)  239 (3.0) |
| **Fertilization method (No.(%))**  IVF  ICSI (including late ICSI) | 2317 (58.5)  1646 (41.5) | 5115 (63.5)  2939 (36.5) |
| **Type of ART procedure (No.(%))**  FET  Fresh | 1810 (45.7)  2153 (54.3) | 5646 (70.1)  2408 (29.9) |
| **Total No. of stimulated cycles (No.(%))**  1  2  ≥3 | 3192 (80.5)  615 (15.5)  157 (4.0) | 6017 (74.7)  1388 (17.2)  649 (8.1) |
| **Period of transferred embryos (No.(%))**  Day 2-3  Day 4  Day 5-6 | 3809 (96.1)  32 (0.8)  122 (3.1) | 7384 (91.7)  48 (0.6)  622 (7.7) |
| **No. of embryos transferred (No.(%))**  1  2  3 | 161 (4.1)  2951 (74.5)  851 (21.5) | 431 (5.4)  7574 (94.0)  49 (0.6) |

†. The year of transplant date
